# The prognostic impact of beta blockers in ischemic heart failure: time for a paradigm shift?

**DOI:** 10.1007/s10741-026-10615-5

**Published:** 2026-03-21

**Authors:** Domenico Simone Castiello, Pierangelo Calvelli, Andrea Anniballo, Letizia Rosa Romano, Alberto Polimeni, Antonio Curcio

**Affiliations:** 1 Department of Pharmacy, Health and Nutritional Sciences, University of Calabria, Rende, CS Italy; 2 Division of Cardiology, Annunziata Hospital, Cosenza, Italy; 3 Department of Advanced Biomedical Sciences, University Federico II, Naples, Italy; 4 Division of Interventional Cardiology, Annunziata Hospital, Cosenza, Italy

**Keywords:** Beta blockers, Optimal medical therapy, Revascularization, Myocardial infarction, Heart failure

## Abstract

Beta-blockers are traditionally considered a cornerstone of the pharmacological therapy in heart failure (HF) post-myocardial infarction (MI) and their use in this setting recognizes a pathophysiological rationale. However, most of evidence supporting their administration in ischemic HF is dated and was conceived before the introduction of modern reperfusion therapies (the so-called “pre-reperfusion era”). The introduction of cutting-edge techniques for revascularization and pharmacological strategies changed the history of MI, reducing mortality and allowing to many patients to overcome the acute event without residual ventricular dysfunction. In the “reperfusion era”, the impact of beta-blocker therapy is different than previously observed and evidence coming from new studies lead to question their role as inevitable drugs after acute events, principally in patients with preserved left ventricular ejection fraction. Consequently, the clinical need of prescribing beta-blockers across the full spectrum of ischemic HF patients has become a debated question. This review aims to retrace the history of beta-blockers in ischemic HF, focusing on recent and ongoing trials, that will offer new and update evidence to guide clinical practice in the next future. Since a “one-size-fits-all” approach cannot be justified, our aim is to propose a tailored approach to each patient’s profile, based on left ventricular ejection fraction, comorbidities, arrhythmic burden and quality of life. Furthermore, we propose an algorithm to guide the therapeutic decision of begin, withhold or withdrawn therapy with beta-blockers from the MI onset to the chronical phase of ischemic HF.

## Introduction

Beta-blockers are cornerstone pharmacologic therapy for numerous diseases including arterial hypertension, coronary artery disease (CAD), cardiac arrhythmias, heart failure (HF), migraine headache, glaucoma, and muscle tremor [1]. Among the major cardiovascular benefits, this prominent class of drugs warrants secondary prevention after myocardial infarction (MI), even more in presence of systolic dysfunction and clinical signs of HF [2]. MI is the leading cause of HF: approximately 70% of patients with HF have CAD and, notably, with up to one-third of patients developing HF after an MI [3]. Noteworthy, improvements in the management of MI together with population ageing have contributed to a growing epidemiological burden of ischemic HF [4]. However, ischemic HF is not a unique entity, and this diagnosis is comprehensive of patients with different clinical, pathophysiological and instrumental features leading to potential selection needed before prescribing beta-blocker therapy [5, 6]. In the context of MI, left ventricular dysfunction may develop even in the absence of overt clinical HF, representing an early stage within the broader continuum that may ultimately lead to ischemic HF [7]. Consequently, several contemporary randomized trials conducted in post-MI populations without symptomatic HF remain highly informative for understanding the evolving role of beta-blocker therapy in the prevention and management of ischemic ventricular dysfunction.

Initially, the use of beta-blockers for ischemic HF was seen as a contraindication, and it took nearly 30 years from the discovery of these drugs to the first clinical trials for use HF. In the 1990’s, some of the earliest beta-blockers’ clinical trials of metoprolol were associated with lower mortality rates [2]. This reinvigorated interest in beta-blockers which proved to reduce cardiovascular (CV)-related hospitalization and death [8]. This line of investigation rapidly gained substantial momentum and scientific appeal, with evidence of 23% reduction in mortality in patients receiving beta-blocker therapy compared with controls at 2-years [9].

This increasingly robust body of evidence led to a strong class of recommendation for the use of beta-blockers in patients with MI and ischemic HF. Currently, beta-blocker therapy is recommended by European Society of Cardiology (ESC) guidelines for all MI patients, regardless of left ventricular ejection fraction (LVEF) [7, 10], and beta-blockers prescription at discharge after MI is considered a performance measure [11]. Otherwise, ESC guidelines have different class of recommendation for beta-blockers in HF, based on the three categories of HF, according to the LVEF value: there is a strong indication only in HF with reduced LVEF (HFrEF; LVEF ≤ 40%), whereas the indication is weaker in patients with HF with mildly reduced LVEF (HFmrEF; LVEF 41–49%) and with preserved LVEF (HFpEF; LVEF ≥ 50%). Beta-blockers are one of the pillars of therapy in patients with HFrEF, together with renin-angiotensin-aldosterone system inhibitors (RAAS-i), mineralcorticoid receptor antagonists (MRAs) and sodium/glucose cotransporter 2-inhibitors (SGLT2i), as they proved to reduce mortality and HF hospitalization [12].

Summarizing, for many years beta-blockers have been prescribed in ischemic HF, regardless of systolic function, and clinical presentations, arrhythmic burden and quality of life [13]. However, most evidence supporting their use and claiming the beneficial effect of this class of drugs in this setting derives from trials dating back many years and specifically in the last two decades of the last century [14–16]. In contemporary clinical practice significant advancements in the management of MI have occurred, encompassing modern reperfusion strategies (including technical advances in percutaneous coronary intervention with modern drug eluting stents, complete revascularization and intracoronary imaging adoption) and modern pharmacological drugs (potent antithrombotic therapies, high-intensity statins along with new lipid-lowering therapies, and RAAS-i) [17–19]. In addition, the introduction of high sensitivity markers allowed early diagnosis of MI, leading to reduced ischemic time for heart [10, 20]. Furthermore, with progresses in the management of MI a growing number of patients overcome acute event with a mild systolic dysfunction or without any systolic damage, raising doubts about real benefit of this class of drugs in post-MI patients with more preserved LVEF. In the last years, results from randomized clinical trials conducted in the “reperfusion era” and even more in the “modern reperfusion era” provided new evidence and lead to reconsider the role of beta-blockers and their impact on mortality and CV outcomes, principally in patients with preserved LVEF [21].

**Fig. 1:**
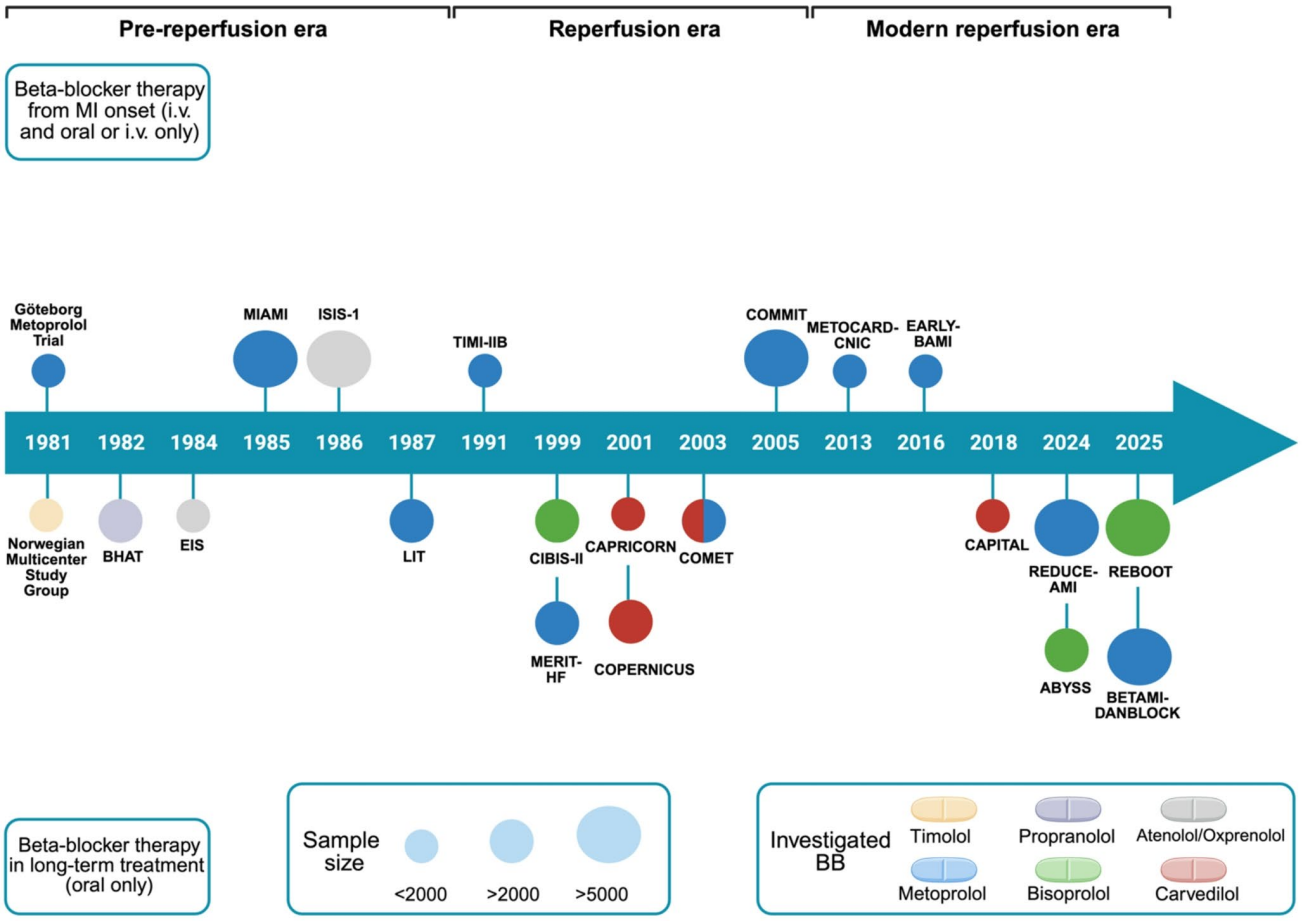
Main clinical trials addressing the use of beta blockers in ischemic heart failure across the last five decades. The size of the circles indicates the magnitude of the studied population. IV: intravenous; MI: Myocardial infarction

The aim of this review is to explore the pathophysiological role of beta-blocker therapy in ischemic HF and MI per se, highlighting the results of trials that have tested beta-blockers in this scenario (Fig. 1). Although not exclusively focused on ischemic HF, we deliberately included landmark trials conducted in the setting of MI, as these studies frequently incorporated HF-related endpoints and provide pivotal insights into disease progression. It should also be acknowledged that several contemporary randomized trials discussed in this review primarily enrolled post-MI patients without overt clinical HF. While these populations differ from patients with established symptomatic HF, they represent an important stage in the ischemic disease continuum, in which therapeutic strategies may influence ventricular remodeling and the subsequent development of HF. Importantly, early initiation of beta-blocker therapy after MI has been consistently associated with a reduced risk of subsequent HF development, thereby directly informing the pathophysiological and therapeutic continuum of ischemic HF. The purpose is, moreover, to discuss the potential change of therapeutic approach in the next future.

## Methods

We performed a narrative review aimed at providing a comprehensive and clinically oriented overview of the evolving role of beta-blocker therapy in patients with ischemic HF in the context of contemporary evidence. A literature search was performed in MEDLINE from database inception to January 2026. The search strategy included combinations of the terms “beta-blockers”, “acute myocardial infarction”, and “ischemic heart failure”. Additional relevant publications were identified through manual screening of reference lists from retrieved articles and key review papers. Studies were considered for inclusion if they investigated beta-blocker therapy in patients with MI or ischemic HF, particularly when reporting outcomes related to HF development, cardiovascular events, or mortality. Particular emphasis was placed on randomized controlled trials, especially those conducted in the contemporary reperfusion era, including the most recent large-scale trials evaluating the role of beta-blockers after MI. In line with the narrative nature of the review, the selection of studies was guided by clinical relevance and contribution to the understanding of the pathophysiological rationale and clinical impact of beta-blocker therapy in ischemic HF. Formal systematic review procedures, including predefined screening flow diagrams, quantitative synthesis, or structured risk-of-bias assessment, were not performed, as they were beyond the scope of this work.

## Pathophysiological rationale of beta-blockers therapy in ischemic heart failure

In patients with ischemic HF, the therapeutic benefit of β-adrenergic receptor blockade arises from the ability to interrupt maladaptive sympathetic activation that links chronic myocardial ischemia to progressive ventricular dysfunction. At the molecular level, sustained β₁-adrenergic stimulation promotes receptor downregulation and desensitization through G-protein–coupled receptor kinase–dependent mechanisms, leading to impaired contractile signaling and loss of adrenergic reserve [22, 23]. Excessive catecholamine exposure further induces calcium overload via hyperactivation of L-type calcium channels and dysfunctional ryanodine receptor phosphorylation, resulting in intracellular calcium leak, mitochondrial injury, and oxidative stress, processes that are particularly deleterious in the metabolically compromised ischemic myocardium [24, 25]. Beta-blockers mitigate these molecular insults by restoring receptor density and signaling fidelity, reducing calcium-dependent cytotoxicity, and improving myocardial energetic efficiency [26, 27]. In ischemic HF, they represent an effective pharmacological strategy, considering their negative inotropic and chronotropic effect mediated by β₁-adrenergic receptors block on cardiomyocytes. By attenuating cardiac contractility and heart rate, beta-blockers reduce excessive myocardial oxygen consumption while preserving stroke volume through improved mechano-energetic coupling, thereby resolving the paradox of negative inotropy translating into long-term functional improvement [28]. Heart rate reduction plays a central role in this process, prolonging diastolic filling and coronary perfusion time, improving myocardial oxygen supply through the increase of subendocardial blood flow in the presence of fixed coronary stenoses, and limiting recurrent ischemic insults that perpetuate contractile dysfunction [29, 30]. Over time, these hemodynamic effects converge with neurohormonal suppression to promote reverse left ventricular remodeling, characterized by reduced chamber dilation, decreased wall stress, and improved systolic efficiency [31, 32]. In the acute MI setting, beta-blockers may also exert protective effects at the microvascular level by reducing myocardial oxygen demand, attenuating sympathetic hyperactivation, and potentially limiting no-reflow injury, mechanisms that may contribute to smaller infarct size and improved ventricular remodeling [33]. These beneficial effects are more pronounced with β₁-selective beta-blockers, such as atenolol, bisoprolol, esmolol, metoprolol, and nebivolol, which, owing to their pharmacodynamic profile, mitigate concerns about potential adverse effects, particularly in patients with respiratory diseases [34]. In addition, beta-blockers contribute to reduce the risk of ventricular potentially life-threatening arrhythmias [35]. They antagonize multiple direct and indirect arrhythmogenic effects of increased sympathetic activity (changes in transmembrane potential homeostasis, nerve remodeling, hypertrophy and fibrosis) and have specific anti-arrhythmic effects (lengthening of the ventricular effective refractory period, suppression of triggered activity and automaticity, slowing of heart rate, and modulation of conduction in re-entrant circuit in the infarct border zone) [35, 36]. Collectively, these molecular and physiological mechanisms provide a coherent pathophysiological rationale for beta-blocker therapy in ischemic HF, explaining how early attenuation of adrenergic-mediated negative inotropy evolves into durable improvements in ventricular structure, function, and clinical outcomes.

## Beta-blockers in the pre-reperfusion era

Although the trials conducted for evaluate the beta-blockers effect in post-MI course in pre-reperfusion era (Table 1) did not specifically enroll patients with established HF, these trials enrolled patients not undergoing reperfusion, hence it can be assumed that they developed at least a mild systolic dysfunction post-MI.

**Table 1:** Main clinical trials conducted in ischemic HF patients during pre-reperfusion era

Trial, Year	Sample size	Beta-blocker evaluated and study design	Timing of therapy	Therapy duration	Primary endpoint	Results
Norwegian Multicenter Study Group [14] (1981)	1,884	Timolol 10 mg bid, oral therapy vs. placebo	Long-term treatment	33 months	All-cause mortality, re-MI	Timolol reduced all-cause mortality (13.3% vs. 21.9%, with a 39.3% reduction, *p* = 0.0003) and re-MI (14.4% vs. 20.1%, with a 28.4% reduction, *p* = 0.0006) compared with placebo
Göteborg Metoprolol Trial [15] (1981)	1,395	Metoprolol 15 mg i.v. once daily followed by 100 mg bid, oral therapy vs. placebo	From MI onset to early phase post-discharge	3 months	All-cause mortality	Metoprolol reduced all-cause mortality (5.7% vs. 8.9%, with a 36% reduction, *p* < 0.03) compared with placebo
BHAT Trial [16] (1982)	3,837	Propranolol 180–240 mg once daily, oral therapy vs. placebo	Long-term treatment	2 years	All-cause mortality	Propranolol reduced all-cause mortality (7.2% vs. 9.8%, with a 26% reduction) compared with placebo
Julian et al. [37] (1982)	1,456	Sotalol 320 mg once daily, oral therapy vs. placebo	Long-term treatment	1 year	All-cause mortality, re-MI	Sotalol reduced all-cause mortality (7.3% vs. 8.9%, with a 18% reduction, but without statistical significance) and re-MI (with a 41% reduction, *p* < 0.05) compared with placebo
Taylor et al. [38] (1982)	1,103	Oxprenolol 40 mg bid, oral therapy vs. placebo	Long-term treatment	6 year	All-cause mortality and cardiac events (death and re-MI)	Oxprenolol did not reduce all-cause mortality (9.5% vs. 10.2%) or cardiac events (18.8% vs. 21.7%) compared with placebo, however a survival beneficial effect was found in patients started the therapy within 4 month from the MI (95.1% vs.76.6%, with a 18.5% difference, *p* < 0.001)
EIS Trial [39] (1984)	1,741	Oxprenolol 160 mg bid, oral therapy vs. placebo	Long-term treatment	1 year	All-cause mortality and cardiac events	In the overall cohort oxprenolol was not associated with a reduction in mortality and cardiac events compared with placebo, on contrary there was a non-significant increase in mortality (6.6% vs. 5.1%)
MIAMI trial [40] (1985)	5,778	Metoprolol 15 mg i.v. once daily followed by 200 mg daily, oral therapy vs. placebo	From MI onset to very early phase post-discharge	15 days	Morbidity and mortality after 15 days	In the overall cohort metoprolol did not reduce mortality rate (4.3% vs. 4.9%, *p* = 0.29) compared with placebo, however a 29% reduction of mortality was found in patients at higher risk of mortality
ISIS-1 [41] (1986)	16,027	Atenolol 5–10 mg i.v. immediately, followed by 100 mg daily, oral therapy vs. placebo	From MI onset to long-term treatment	1 year	Vascular mortality	Atenolol significantly reduced vascular mortality (10.7% vs. 12.0%, *p* < 0.01) compared with placebo
LIT [42] (1987)	2,395	Metoprolol 100 mg bid, oral therapy vs. placebo	Long-term treatment	1 year	All-cause mortality	Metoprolol did not reduce all-cause mortality (65 deaths vs. 62 deaths) compared with placebo, but the study was under-powered
Herlitz et al. [43] (1988)	1,395	Metoprolol 200 mg daily, oral therapy for 3 months vs. placebo	Long-term treatment	5 years	All-cause mortality	Metoprolol therapy for 3 months reduced mortality rates at 3 months and 2 years, but not at final 5-years follow-up (24.2% vs. 25.7%, *p* > 0.2)

*BID* bis in die, *IV* intravenous, *MI *myocardial infarction

In the Norwegian Multicenter Study Group, 1,884 surviving MI patients were randomized (7 to 28 days after MI) to long-term treatment with timolol (oral 10 mg twice daily) or placebo. In this cohort, timolol proved to reduce mortality and rate of reinfarction by 39.3% and 28.4%, respectively at a median follow-up of 17 months [14].

In the same year, the Göteborg Metoprolol Randomized Trial was published. In 1,395 patients with definite or suspected MI, the treatment with Metoprolol (15 mg i.v. followed by oral administration of 100 mg twice daily) lead to a 36% reduction in the primary endpoint of all-cause mortality compared with placebo [15].

The BHAT (Beta-blocker Heart Attack Trial) trial was a randomized, double-blind, placebo-controlled trial in which 3,837 patients with recent MI (5–21 days) were randomized to either daily propranolol hydrochloride or placebo during a 27-months interval. Total mortality was reduced in propranolol group by 26% and so were arteriosclerotic heart disease and sudden cardiac death. Authors underlined that the trial was not designed to answer to the question how long after MI beta blocker should be administered, but beneficial effect of beta-blockers seemed to be sustained for the duration of the trial (an average of 25 months) [16].

In 1982 two different beta-blockers were evaluated in two randomized trials. Julian DG et al. conducted a randomized study for assessing the potential benefit of sotalol in 1,456 patients after MI (5–14 days). Sotalol was associated with a non-significant reduction of all-cause mortality, whereas a 41% significant reduction of reinfarction was found compared with placebo [37]. Oxprenolol was evaluated in long-term (7-years) therapy in a cohort of 1,103 MI patients who were randomized to oxprenolol 40 mg twice daily or placebo between 1 and 90 months previously. This drug did not provide any significant difference in mortality or cardiac events. However, the time at which treatment was started had a significant impact. Indeed, for the 417 patients who received the experimental drug within four months from the MI, oxprenolol reduced significantly the mortality rate, whereas this beneficial effect was not observed in those starting the therapy between 5 and 12 month after the MI and, on contrary, the survival rate was reduced in patients who started oxprenolol more than a year after the MI compared with placebo, with an unexpected harmful finding [38]. Oxprenolol therapy was also investigated in the EIS (European Infarct Study) trial which enrolled 1,741 patients 14–36 days post-MI. In this population oxprenolol therapy was associated with a non-significant trends toward increase mortality compared with placebo. Interestingly, concerns of a significant increased mortality emerged in some subgroups: in patients who discontinued the therapy, in patients older than 65 years and in patients at low risk of ischemic events [39].

In the MIAMI (Metoprolol In Acute Myocardial Infarction) trial, 5,778 patients with definite or suspected MI were randomized to i.v. metoprolol (15 mg) or placebo. Metoprolol was administered early after patient’s arrival to hospital and within 24 h after symptoms onset. Then oral treatment with metoprolol 200 mg daily was continued for 15 days. In the overall cohort no differences in mortality were revealed. Notably, a subgroup of patients showed higher risk of mortality and contained approximately 30% of all randomized patients. In these, the mortality rate in the metoprolol treated group was 29% less than in the placebo group [40].

ISIS-1 (First International Study of Infarct Survival Collaborative Group) trial is one of the largest studies belonging to the “pre-reperfusion era”. Between 1981 and 1985, 16,027 patients with suspected MI (at a mean of 5 h after symptoms onset) were randomized either to placebo or to receive intravenous atenolol (5–10 mg immediately), followed by 100 mg/day orally for 7 days. During the treatment period (days 0–7), vascular mortality was significantly lower in the treated group (3.89% vs. 4.57%, *p* < 0.04). After the treatment period, only a slight divergence was observed so that vascular mortality remained significantly lower in the atenolol group at one year (10.7% vs. 12.0%, *p* < 0.01). Most of the improvements in vascular mortality were seen during days 0–1, despite immediate beta-blockade increased the extent of inotropic drug use [41].

In the LIT (Lopressor Intervention Trial) trial 2,395 patients with recent MI (6–16 days) were randomized to oral metoprolol 100 mg bid or placebo. After 1 year, there was no difference in mortality rate, but the study was prematurely terminated because of a decline in patient enrollment. Hence it was under-powered for detecting mortality reduction [42]. Finally, Herlitz et al. found a significant reduction of mortality in 1,395 patients with suspected MI randomized to 3-months therapy with metoprolol compared with placebo until 2-years follow-up, but not at 5-years [43].

## Beta blockers in the reperfusion era

The reperfusion era began between 1976 and 1977 with respectively the first reports of intracoronary thrombolysis for the treatment of MI and the first percutaneous coronary intervention (PCI), performed by Dr. Andreas Grüntzig. However, the introduction of i.v. thrombolysis into widespread clinical practice occurred in 1980s and only in 1993 evidence showed that primary PCI outperformed thrombolysis for acute reperfusion therapy [44–46]. Since the introduction of revascularization strategies, the prognosis of MI patients changed dramatically, with a significant improvement in survival rate, consistently with a reduction in recurrent ischemic events, with an established evidence of superior efficacy for primary PCI compared with fibrinolysis [47, 48]. In this respect, the trials conducted in this era for investigate the beneficial effect of beta-blockers in MI patients are closer to our current clinical practice and have a greater scientific weight (Table 2).

**Table 2:** Main clinical trial in ischemic HF patients during reperfusion era

Trial, Year	Sample size and cohort features	Reperfusion strategy	Beta-blocker evaluated and study design	Timing of therapy	Therapy duration	Primary endpoint	Results
TIMI-II B Trial [49] (1991)	1,434 (100% STEMI)	100% Fibrinolysis	Immediate (15 mg i.v., followed by oral 50 mg bid on day 1 then 100 mg bid) vs. deferred (oral 50 mg bid on day 6 then 100 mg bid) metoprolol	From MI onset to discharge	6 days	LVEF at the time of hospital discharge assessed invasively	LVEF and mortality were similar between the two groups, whereas immediate therapy reduced the re-MI rate and recurrent chest pain
CIBIS-II Trial [51] (1999)	2,647 (50% ischemic HF, LVEF ≤ 35%)	Unknown	Bisoprolol (initial 1.25 mg oral daily followed by uptitration to a maximum of 10 mg daily) vs. placebo	Long-term treatment	1.3 years	All-cause mortality	Bisoprolol significantly reduced all-cause mortality (11.8% vs. 17.3%, HR: 0.66; 95% CI: 0.54–0.81, *p* < 0.0001) compared with placebo
MERIT-HF Trial [32] (1999)	3,991 (65% ischemic HF, LVEF ≤ 40)	Unknown	Metoprolol 12.5–25 mg until 200 mg oral daily vs. placebo	Long-term treatment	1 year	All-cause mortality	Metoprolol reduced mortality (7.2% vs. 11.0%, RR: 0.66; 95 CI: 0.53–0.81, *p* = 0.00009) compared with placebo
CAPRICORN Trial [52] (2001)	1,959 (100% ischemic HF, LVEF ≤ 40%)	46% Fibrinolysis or primary PCI	Carvedilol (initial 6.25 mg daily bid followed by uptitration to a maximum of 25 mg bid oral) vs. placebo	Long-term treatment	1.3 years	All-cause mortality or CV hospital admissions	Carvedilol did not reduce the primary endpoint (35% vs. 37%, HR: 0.92; 95 CI: 0.80–1.07) compared with placebo, but reduced all-cause mortality and MI
COPERNICUS Trial [31] (2001)	2,289 (67% ischemic HF, LVEF ≤ 25%)	Unknown	Carvedilol (initial 3.125 mg bid oral followed by uptitration to a maximum of 25 mg bid) vs. placebo	Long-term treatment	10.4 months	All-cause death and composite of death, or admission for any cause	Carvedilol significantly reduced mortality (by 35%; *p* = 0.00013) compared with placebo
COMET Trial [53] (2003)	3,029 (53% ischemic HF, LVEF ≤ 35%)	Unknown	Carvedilol (target dose 25 mg twice daily oral) vs. Metoprolol (target dose 50 mg twice daily oral)	Long-term treatment	4 years	All-cause death and composite of death, or admission for any cause	Carvedilol significantly reduced mortality (34% vs. 40%, HR: 0.83; 95% CI: 0.74–0.93, *p* = 0.0017) compared with metoprolol
COMMIT Trial [54] (2005)	45,852 (93% STEMI, 7% NSTEMI, 24% HF)	54% Fibrinolysis	Metoprolol (15 mg i.v. then 200 mg oral daily) vs. placebo	From MI onset to long-term treatment	1 month	All-cause death and composite of death, re-MI, or cardiac arrest	Metoprolol was not superior in terms of all-cause death (7.7% vs. 7.8%, OR: 0.99; 95% CI: 0.92–1.05, *p* = 0.69) nor in the composite outcome (9.4% vs. 9.9%, OR: 0.96; 95% CI: 0.90–1.01) compared with placebo

*CV* cardiovascular, *HF* Heart failure, *LVEF* Left ventricular ejection fraction, *MI* Myocardial infarction, *NSTEMI* Non-ST-elevation MI, *PCI* percutaneous coronary intervention, *STEMI* ST-elevation MI

The TIMI-IIB (Thrombolysis in Myocardial Infarction IIB) trial was one of the first trial investigating the role of beta-blockers administration in patients treated with a reperfusion therapy (100% fibrinolysis). 1,434 patients with ST-elevation MI (STEMI) and treated with rt-PA < 4 h after the onset of symptoms were randomized to immediate (3 doses of 5 mg i.v., followed by 50 mg bid on day 1 then 100 mg bid) or deferred metoprolol (oral metoprolol 50 mg bid on day 6 followed by 100 mg bid thereafter). The study showed that metoprolol was safe when given early after thrombolytic therapy and it was associated with decreased myocardial ischemia and reinfarction in the first week but offer no benefit over late administration either in improving LVEF at discharge or in reducing mortality [49].

Van de Werf F et al. compared atenolol (5–10 mg i.v. followed by 25–50 mg orally bid) therapy with a specific bradycardic agent (alinidine) and with placebo in 292 patients with MI undergoing fibrinolysis in a randomized trial. In this cohort no significant differences in left ventricular function, coronary artery patency, and infarct size were revealed among the three groups [50].

Bisoprolol was evaluated for the first time in HF in the CIBIS-II (The Cardiac Insufficiency Bisoprolol Study II) trial which enrolled 2,647 HF patients with a LVEF ≤ 35% (of whom 50% had ischemic HF). Bisoprolol (administrated orally from 1.25 mg daily to a maximum of 10 mg daily) reduced significantly the primary endpoint of all-cause mortality at a mean follow-up of 1.3 years compared with placebo (11.8% vs. 17.3%, hazard ratio [HR]: 0.66; 95% confidence intervals [CI]: 0.54–0.81, *p* < 0.0001). Furthermore, a significant reduction of sudden deaths was found. Notably, the benefits were consistent in all subgroups, without differences based on severity or cause of HF [51].

The MERIT-HF (Metoprolol CR/XL Randomised Intervention Trial in-Congestive Heart Failure) trial enrolled 3,991 HF patients with a LVEF ≤ 40% (65% with ischemic etiology) who were randomized to Metoprolol controlled release/extended release 12.5–25 mg oral once daily (with a target dose of 200 mg once daily) or placebo. At 1-year Metoprolol proved superiority in reducing all-cause mortality (7.2% vs. 11.0%, HR: 0.66; 95% CI: 0.53–0.81, *p* = 0.00009), but also reduce sudden deaths and death from worsening HF [32].

In the CAPRICORN (Carvedilol Post-Infarct Survival Control in LV Dysfunction) trial 1,959 patients with recent MI and left ventricular dysfunction (LVEF ≤ 40%) were randomized to carvedilol 6.25 mg (up titrated to the highest tolerated dose) or placebo and followed up for a mean of 1.3 years. Patients were on treatment with ACE-inhibitors, as well, unless contraindicated. Reperfusion therapy, mainly by thrombolysis but also by primary PCI, was applied in 46% of all patients. Carvedilol did not reduce the primary endpoint of all-cause mortality of CV hospital admissions, but it did show a significant reduction of all-cause mortality alone and the incidence of recurrent, non-fatal MI alone. The reduction in all-cause mortality was additional to the effects of ACE-inhibitors and reperfusion therapy, underlining that patients with left-ventricular dysfunction after MI remain at high risk despite the benefits provided by modern therapies [52].

The COPERNICUS (Carvedilol Prospective Randomized Cumulative Survival) trial enrolled 2,289 HF patients with a LVEF ≤ 25% (67% with ischemic HF). The experimental strategy was the administration of carvedilol (initial 3.125 mg bid oral followed by uptitration to a maximum of 25 mg bid) in comparison with placebo. Long-term treatment with carvedilol significantly reduced the all-cause mortality rate (by 35%; *p* = 0.00013) and the composite of deaths or hospitalization (by 24%, *p* < 0.001). This benefit was consistent in patients with recent or recurrent HF decompensation [31].

A direct head-to-head comparison between different beta-blockers was conducted in the COMET (Carvedilol Or Metoprolol European Trial) trial. 3,029 HF patients with a LVEF ≤ 35% (53% ischemic HF) were randomized to carvedilol (target dose 25 mg twice daily) or metoprolol (target dose 50 mg twice daily). Long-term treatment with carvedilol significantly reduced the all-cause mortality rate (34% vs. 40%, HR: 0.83; 95% CI: 0.74–0.93, *p* = 0.0017) but not the composite of deaths or hospitalization (74% vs. 76%, HR: 0.94; 95% CI: 0.86–1.02, *p* = 0.122) compared with metoprolol at 4-years [53].

In the COMMIT (Clopidogrel and Metoprolol in Myocardial Infarction Trial) trial, 45,852 MI patients (of whom 24% were admitted with HF) were randomized to immediate i.v. metoprolol therapy within 24 h after the onset of suspected MI followed by oral medication for 28 days or placebo. The metoprolol strategy did not reduce all-cause mortality (7.7% vs. 7.8%, OR: 0.99; 95% CI: 0.92–1.05, *p* = 0.69) neither the composite of death, re-MI, or cardiac arrest (9.4% vs. 9.9%, OR: 0.96; 95% CI: 0.90–1.01). However, metoprolol proved to reduce the risks of re-MI and ventricular fibrillation. An increase of the risk of cardiogenic shock was observed, mainly during days 0–1 after admission, thus leading investigators to recommend beta-blockers only after hemodynamic stabilization after MI [54]. To summarize, data from the pre-reperfusion and reperfusion eras were pooled in a wide meta-analysis including 16 randomized trials with 102,003 patients and which was performed stratifying trials into pre-reperfusion-era or reperfusion-era (> 50% undergoing reperfusion or receiving aspirin/statin) trials. Beta-blocker therapy significantly reduced all-cause mortality in the former group (incident rate ratio [IRR]:0.86; 95% CI: 0.79–0.94), but not in the reperfusion trials (IRR: 0.98; 95% CI: 0.92–1.05) [21].

## Beta blockers in the modern reperfusion era: cutting-edge evidence

The modern reperfusion era includes trials conducted when several currently adopted standard of care (in terms of PCI and medical therapy, with high rates of DAPT, statins and RAAS-i) were already available in practical clinical care. For this reason, even with a gradually advancement of care overtime, these trials (Table 3) represent the most impactful evidence in actual clinical practice.

**Table 3:** Main clinical trial in ischemic HF patients during modern reperfusion era

Trial, Year	Sample size	Reperfusion strategy	Beta-blocker evaluated and study design	Timing of therapy	Therapy duration	Primary endpoint	Results
METOCARD- CNIC Trial [55] (2013)	270 (100% anterior STEMI)	100% PCI	Metoprolol (up to three 5 mg bolus i.v. followed by oral administration) vs. placebo	From MI onset to a very early phase	5–7 days	Infarct size evaluated by MRI 5 to 7 days post-STEMI	Metoprolol significantly reduced the infarct size (25.6 ± 15.3 vs. 32.0 ± 22.2, *p* = 0.012) compared with placebo
EARLY-BAMI Trial [56] (2016)	683 (100% STEMI)	100% PCI	Metoprolol (two 5 mg bolus i.v. followed by oral administration) vs. placebo	From MI onset to a very early phase post-discharge	30 days	Infarct size (% of LV) evaluated by MRI 30 days post-STEMI	Metoprolol was not superior in reducing infarct size (15.3 ± 11.0% vs. 14.9 ± 11.5%, *p* = 0.616) compared with placebo
CAPITAL-RCT [57] (2018)	801 (100% STEMI, LVEF ≥ 40%)	100% PCI	Carvedilol (maximum dose of 20 mg daily, oral) vs. placebo	Long-term treatment	3.9 years	Composite of all-cause death, MI, hospitalization for ACS and HF	Carvedilol was not superior to placebo (6.8% vs. 7.9%, *p* = 0.20)
REDUCE-AMI Trial [59] (2024)	5,020 (35% STEMI, 65% NSTEMI, LVEF ≥ 50%)	95% PCI, 4% CABG	Beta-blocker therapy (metoprolol 62% or bisoprolol 38%) vs. placebo	Long-term treatment	3.5 years	Composite of all-cause death or new MI	Beta-blocker therapy was not superior to placebo (7.9% vs. 8.3%, HR: 0.96; 95% CI: 0.79–1.16, *p* = 0.64)
ABYSS Trial [61] (2024)	3,698 stable CCS (63% previous STEMI, 37% previous NSTEMI, LVEF ≥ 40%)	> 95% PCI, < 5% CABG	Beta-blocker therapy withdrawal (Bisoprolol 80%, or others 20%) vs. continuation	Long-term treatment	3 years	Composite of death, MI, stroke, or hospitalization for CV causes	Beta-blocker therapy withdrawal was not non-inferior to continuation (23.8% vs. 21.1%, HR: 1.16; 95% CI: 1.01–1.33, *p* = 0.44 for non-inferiority)
REBOOT Trial [62] (2025)	8,505 (51% STEMI, 49% STEMI, LVEF ≥ 40%)	94% PCI, 0.2% CABG	Beta-blocker therapy (Bisoprolol 86% or others 14%) vs. placebo	Long-term treatment	3.7 years	Composite of death from any cause, MI, or hospitalization for HF	Beta-blocker therapy was not superior to placebo (HR: 1.04; 95% CI: 0.89–1.22, *p* = 0.63)
BETAMI-DANBLOCK Trial [64] (2025)	5,574 (48% STEMI 52% NSTEMI, LVEF ≥ 40%)	93% PCI, 2% CABG	Beta-blocker therapy (Metoprolol 95%) vs. placebo	Long-term treatment	3.5 years	Composite of death from any cause, MI, coronary reperfusion, stroke, HF or malignant arrhythmias	Beta-blocker therapy was superior to placebo (14.2% vs. 16.3%, HR: 0.85; 95% CI: 0.75–0.98, *p* = 0.03)
Meta-analysis of CAPITAL, REBOOT, and BETAMI-DANBLOCK [65] (2025)	1,885 (68% STEMI, 32% NSTEMI, LVEF 40–49%)	95% PCI, 1% CABG	Beta-blocker therapy (Metoprolol 49%, Bisoprolol 44%, or others 7%) vs. placebo	Long-term treatment	3.5 years	Composite of all-cause death, new MI, or HF	Beta-blocker therapy was superior to placebo (11% vs. 14%, HR: 0.75; 95% CI: 0.58–0.97, *p* = 0.031)
Meta-analysis of CAPITAL, REDUCE-AMI, REBOOT, and BETAMI-DANBLOCK [66] (2025)	17,801 (45% STEMI, 55% NSTEMI, LVEF ≥ 50%)	94% PCI, 2% CABG	Beta-blocker therapy (Bisoprolol 47%, Metoprolol 46%, or others 7%) vs. placebo	Long-term treatment	3.6 years	Composite of all-cause death, MI, or HF	Beta-blocker therapy was not superior to placebo (8.1% vs. 8.3%, HR: 0.97; 95% CI: 0.87–1.07, *p* = 0.54)

*ACS* Acute coronary syndromes, *CCS *Chronic coronary syndrome, *CV *cardiovascular, *HF* Heart failure, *LV* left ventricle, *MI *Myocardial infarction, *MRI *Magnetic resonance imaging, *NSTEMI* Non-ST-elevation MI, *PCI* Percutaneous coronary intervention, *STEMI* ST-elevation MI

The METOCARD-CNIC (The Effect of Metoprolol in Cardioprotection During an Acute Myocardial Infarction) trial was designed to randomly investigate the effect of beta-blockers on infarct size when administered i.v. before reperfusion through PCI in 270 patients with anterior STEMI and infarct size at magnetic resonance performed at 5–7 days after MI was selected as primary endpoint. The study showed that early i.v. metoprolol before PCI significantly reduced infarct size at magnetic resonance and increased LVEF with no excess of adverse events during the first 24 h after STEMI [55].

Early administration of i.v. metoprolol was also investigated in the EARLY-BAMI (Early-Beta blocker Administration before reperfusion primary PCI in patients with ST-elevation Myocardial Infarction) trial which enrolled 683 STEMI patients. In this cohort, metoprolol was not superior in reducing infarct size (% of left ventricle) at magnetic resonance performed at 30 days post-STEMI (15.3 ± 11.0% vs. 14.9 ± 11.5%, *p* = 0.616) and no differences were observed in terms of LVEF [56].

The potential beneficial effect of carvedilol in modern reperfusion era was assessed in the CAPITAL (Carvedilol Post-Intervention Long-Term Administration in Large-scale) trial, in which 801 STEMI patients with successful primary PCI and with LVEF ≥ 40% were randomly assigned either to carvedilol (with a maximum dose of 20 mg daily, within 24 h) or to no beta-blocker. During median follow-up of 3.9 years, the cumulative 3-year incidences of both the primary composite endpoint of all-cause death, MI, hospitalization for HF and for ACS (6.8% vs. 7.9%, *p* = 0.20) and any coronary revascularization (20.3% vs. 17.7%, *p* = 0.65) were not significantly different between the carvedilol and no beta-blockers groups. Also, no significant difference in LVEF at 1-year between the 2 groups was noted (60.9 ± 8.4% and 59.6 ± 8.8%, *p* = 0.06). The result was consistent across the full spectrum of LVEF [57]. However, a beneficial effect was found the 280 patients with LVEF 40–55% with a significant reduction of the secondary composite outcome of cardiac death, MI, and hospitalization for HF (HR: 0.32; 95% CI: 0.10–0.99, *p* = 0.047) at 3.7 years [58].

Following the CAPITAL trial, interest for beta-blocker therapy in ischemic HF has been renewed and four randomized trials have been conducted, designed to investigate its role in post-MI patients with mildly reduced or preserved LVEF, filling a major gap previously limited to observational studies.

The REDUCE-AMI (Randomized Evaluation of Decreased Usage of Beta-BloCkerEs after Acute Myocardial Infarction) trial enrolled 5,020 MI patients with a LVEF ≥ 50% who were randomized to either long-term beta-blocker treatment (metoprolol, 62% or bisoprolol, 38%) or no beta-blocker treatment within 7 days after the MI. After a median follow up of 3.5 years, beta-blocker therapy was not superior to placebo (7.9% vs. 8.3%, HR: 0.96; 95% CI: 0.79–1.16, *p* = 0.64) in terms of primary composite endpoint of death from any cause or new MI. In addition, beta-blocker treatment was not related to a lower incidence of secondary endpoints (death from any causes, death from CV causes, MI, hospitalization for atrial fibrillation or for HF) [59]. In a pre-specified sub-study from REDUCE-AMI trial, effects of beta-blockers on self-reported quality of life (QoL) were investigated using EuroQol 5-Dimension (EQ-5D) and World Health Organization well-being index-5 (WHO-5) questionnaires, obtained at 6–10 weeks and 11–13 months after MI. No differences has been reported in patients on long-term beta-blockers therapy, challenging the traditional idea that withdrawal of this therapy is justified by intolerance [60].

The optimal duration of treatment with beta-blockers after uncomplicated MI is a controversial aspect, as well. The ABYSS (Assessment of Beta-Blocker Interruption 1 Year after an Uncomplicated Myocardial Infarction on Safety and Symptomatic Cardiac Events Requiring Hospitalization) trial tried to provide evidence in this respect, randomizing 3,698 patients with history of MI (the median time between the last MI and randomization was 2.9 years) with a LVEF ≥ 40% to long-term beta-blocker therapy interruption or continuation (80% bisoprolol). The primary endpoint was a composite of death, nonfatal MI, nonfatal stroke, or hospitalization for CV reason. The main secondary endpoint was the change in QoL as measured by the EQ-5D questionnaire. Interruption of long-term beta-blocker treatment was not found to be noninferior to a strategy of beta-blockers continuation (23.8% vs. 21.1%, HR: 1.16; 95% CI: 1.01–1.33; *p* = 0.44) at 3 years, primarily for a lower rate of CV hospitalizations in the continuation group. Importantly, no clear differences were observed in hard outcomes such as death or myocardial infarction, suggesting that these findings should be interpreted cautiously when considering treatment withdrawal in selected patients. Furthermore, beta-blocker discontinuation was not associated with a QoL improvement in the enrolled patients [61].

More recently, the REBOOT (Treatment with Beta-Blockers after Myocardial Infarction without Reduced Ejection Fraction) trial tested the benefits of beta-blocker therapy (bisoprolol in 86% of cases) compared with no beta-blocker therapy in 8,505 patients discharged after MI and with an LVEF ≥ 40%. At 3.7 years follow-up, beta-blockers showed no beneficial effect in terms of primary composite endpoint of death from any cause, re-MI, or hospitalization for HF (HR: 1.04; 95% CI: 0.89–1.22, *p* = 0.63). More deeply, some subgroup effect were revealed: there was an harmful effect of beta-blocker therapy in both women compared with men and in STEMI patients compared with those with NSTEMI, whereas a positive effect was found in patients with LVEF 40–49%, who, however, were only the 10% of the total cohort [62]. Of note, a post-hoc analysis of the REBOOT trial demonstrated that neither withholding (maintenance of drug non-use) nor withdrawal (in patients on beta-blocker therapy before MI) were associated with increased short-term or recurrent ischaemic events, confirming the findings of the main trial in the former scenario and alleviate concerns about a potential ischemic rebound effect in the latter [63].

The combined BETAMI-DANBLOCK (Norwegian Beta-Blocker Treatment after Acute Myocardial Infarction in Revascularized Patients without Reduced Left Ventricular Ejection Fraction - Danish Trial of Beta-Blocker Therapy after Myocardial Infarction without Heart Failure) trial randomized 5,574 MI patients (within 14 days of MI) with LVEF ≥ 40% to beta-blocker therapy (metoprolol in 95% of cases) or no-beta-blocker therapy. The primary endpoint was a composite of death from any cause, MI, coronary revascularization, stroke, HF or malignant arrhythmias. At 3.5 years of follow-up, beta-blocker therapy significantly reduced the primary endpoint rate (14.2% vs. 16.3%, HR: 0.85; 95% CI: 0.75–0.98, *p* = 0.03) compared with no therapy with beta-blockers, mainly driven by a reduction in MI rates [64].

Considering their discordant findings, the data from these trials, which enrolled more than 23,000 patients across 10 countries, were quickly pooled in two individual patient-level meta-analyses. The first one selectively enrolled patients with LVEF 40–49% in whom a signal of benefit emerged from the original trials. This meta-analysis included 1,885 MI patients from CAPITAL, REBOOT and BETAMI-DANBLOCK trials and confirmed the feelings: beta-blocker therapy (mainly with metoprolol, in 49%, and bisoprolol in 44%) significantly reduced the primary composite endpoint of all-cause death, new MI or HF (11% vs. 14%, HR: 0.75; 95% CI: 0.58–0.97, *p* = 0.031) at 3.5 years. Noteworthy, this meta-analysis found a weak but statistically significant interaction between age and the benefits of beta-blockers: patients younger than 75 years appeared to benefit from beta-blocker therapy, while those aged 75 years or older did not [65]. The second meta-analyses was, on contrary, centered on preserved LVEF. For this purpose, the 17,801 patients with LVEF ≥ 50% were included from CAPITAL, REDUCE-AMI, REBOOT and BETAMI-DANBLOCK trials. In this population beta-blocker therapy (mainly with bisoprolol, 47%, and metoprolol 46%) had no benefits in terms of all-cause death, MI, or HF (8.1% vs. 8.3%, HR: 0.97; 95% CI: 0.87–1.07, *p* = 0.54) at a mean follow-up of 3.6 years. Notably, findings were consistent across all subgroups, without evidence of harmful effect in women and in STEMI patients, as revealed in the REBOOT trial [66].

## Therapeutic perspectives and future directions of guidelines

Since 1980s, many progresses have been obtained in the management of patients with MI, considering early diagnosis, pharmacological therapy and interventional treatment. It may be reasonable to think that the impact of the same therapy could be very different across nearly five decades.

The recent generation of randomized trials, including CAPITAL, REDUCE-AMI, ABYSS, REBOOT, and BETAMI-DANBLOCK, has profoundly reshaped the contemporary understanding of beta-blocker therapy in ischemic HF in the era of systematic revascularization and optimized background pharmacotherapy [57, 59, 61, 62, 64]. The overall finding is that beta-blocker therapy is beneficial in patients with ischemic HF and mildly reduced LVEF, whereas, in patients with preserved LVEF, no effect was found, consistently with previously published observational and meta-analyzed data. This is not unexpected, considering that LVEF is a strong prognostic factor both in patients with MI and in those with HF [30, 67]. The relationship between beta-blockers impact on mortality and LVEF could be attributed to pathological substrate of reduced LVEF. In hearts with systolic dysfunction a large amount of non-viable and extensive scarred tissue is present, providing a substrate for re-entrant circuits and fatal ventricular arrhythmias, against which beta-blockers are efficacious. In heart with preserved LVEF, the burden of myocardial injury and infarct-related scar is small, with a subsequent decreased vulnerability to ventricular arrhythmias. Furthermore, patients with preserved LVEF have generally fewer comorbidities and a better prognosis. Consequently, pharmacological effects of beta-blockers may be less relevant in this population [21].

Collectively, these studies invite a careful re-evaluation of the long-standing practice of routine and indefinite beta-blocker prescription after MI, particularly in patients without reduced LVEF. Although the apparent heterogeneity of trial results may initially seem difficult to reconcile, closer examination suggests that much of this variability reflects differences in study design and endpoint definitions. REDUCE-AMI and REBOOT were primarily designed to evaluate whether routine long-term beta-blocker therapy provides prognostic benefit in contemporary wide cohort of post-MI patients with preserved or mildly reduced LVEF treated with modern reperfusion and guideline-directed medical therapy [59, 62]. In contrast, BETAMI-DANBLOCK enrolled a more selected population with less comorbidities (with lower rates of arterial hypertension, diabetes and dyslipidemia), but most importantly, adopted a different primary endpoint [64]. Indeed, while REDUCE-AMI focused on a relatively strict composite of all-cause death or recurrent MI, and REBOOT included death, MI, or HF hospitalization, the primary endpoint in BETAMI-DANBLOCK was broader, encompassing death, MI, coronary revascularization, stroke, HF, or malignant arrhythmias. As a result, differences in event composition may have contributed to the modest treatment effect observed in BETAMI-DANBLOCK. In this respect, trials adopting a “prospective” strategy, hence randomizing patients early after the index infarction (CAPITAL, REDUCE-AMI, REBOOT, BETAMI-DANBLOCK), consistently failed to demonstrate a reduction in “hard” endpoints such as all-cause mortality, re-MI, or new-onset HF among patients with preserved LVEF. In contrast, ABYSS, with its “retrospective” non-inferiority design focused on beta-blockers withdrawal after long-term use, yielded positive results driven predominantly by “softer” endpoints, including CV rehospitalizations. This distinction is crucial, as soft endpoints are inherently more susceptible to adjudication bias in open-label trials, even when PROBE methodologies are employed [68]. Importantly, when analyses are restricted to hard clinical outcomes such as death, recurrent myocardial infarction, or HF-related hospitalization, the available evidence from contemporary randomized trials generally suggests a limited incremental prognostic benefit in revascularized patients with preserved LVEF and no history of HF. Conversely, patients with mildly reduced LVEF (40–49%) appear to represent a biologically distinct subgroup, pathophysiologically near to patients with reduced LVEF, a pattern already observed in HF [69, 70]. Additional support for the concept of HFmrEF as a distinct clinical phenotype also emerges from real-world observational data. In a multicenter registry analysis, patients with mildly reduced LVEF were shown to display clinical characteristics intermediate between those of HFrEF and HFpEF populations, including a higher prevalence of CAD and a more frequent use of beta-blocker therapy in routine clinical practice [71]. Although observational in design, these findings provide complementary real-world evidence suggesting that clinicians often manage HFmrEF patients with therapeutic strategies closer to those adopted for HFrEF, further supporting the rationale for considering this subgroup separately within the spectrum of post–myocardial infarction patients. In the individual patient-level meta-analysis pooling data from REBOOT, BETAMI-DANBLOCK, and CAPITAL trial, which included 1,885 patients with LVEF between 40% and 49%, beta-blocker therapy was associated with a significant reduction in the composite endpoint of all-cause death, MI, or HF (11% vs. 14%, HR: 0.75; 95% CI: 0.58–0.97) at a median follow-up of approximately 3.5 years. This corresponds to an absolute risk reduction of about 3%, translating into an estimated number needed to treat of roughly 30–35 patients to prevent one major cardiovascular event during follow-up [65]. Although modest in absolute terms, this magnitude of benefit is clinically meaningful when considered in the context of a widely available, low-cost therapy with a well-established safety profile. Interaction analyses from the same dataset al.so suggested that the treatment effect may vary across certain patient characteristics. In particular, a weak but statistically significant interaction with age was observed, with patients younger than 75 years appearing to derive greater benefit from beta-blocker therapy than older individuals. Conversely, no consistent interaction was reported according to sex or infarct type (STEMI versus NSTEMI), although the statistical power for these subgroup analyses was limited. Taken together, these findings reinforce the concept that patients with mildly reduced LVEF may represent an intermediate-risk population that remains sufficiently vulnerable to adverse remodeling and arrhythmic events requiring a management strategy more similarly to those with overt systolic dysfunction [65]. Another relevant aspect concerns the type and timing of beta-blocker initiation. While bisoprolol was the most commonly used beta-blocker in the REBOOT and ABYSS trials, metoprolol was more frequently prescribed in the BETAMI-DANBLOCK and CAPITAL-RCT studies, with dosing left to the discretion of the investigators. Specific pharmacological properties, such as the greater β1-selectivity and longer half-life of bisoprolol (with a lower likelihood of adverse effects in patients with respiratory disease), or the superior efficacy of metoprolol in the treatment of myocardial ischemia and angina (a possible explanation for the lower rate of reinfarction observed in the BETAMI-DANBLOCK study), may contribute to the significant differences observed among the various studies, thereby raising questions about the existence of a true class effect. About the timing, in CAPITAL-RCT and REDUCE-AMI treatment was initiated earlier (within 7 days) than REBOOT and BETAMI-DANBLOCK (within 14 days) after the index event. Differences in treatment exposure and adherence over time may also have contributed to variability in outcomes. In this respect, the adherence was higher in BETAMI-DANBLOCK (88.6% at 6-months) compared with REDUCE-AMI (81.9% at 11–13 months) and REBOOT (55.3% at 48 months). Finally, differences in statistical power and event rates should also be considered. In relatively low-risk contemporary populations with preserved LVEF, event rates are inherently low, making it challenging to detect modest treatment effects. Consequently, even large trials may be underpowered to identify small but potentially clinically relevant differences in outcomes. Also, it should be noted that BETAMI-DANBLOCK trial derives from the ongoing combination of two different trials (BETAMI and DANBLOCK) which were not completed separately because of low enrollment and a low incidence of events.

Taken together, these considerations suggest that the findings of REDUCE-AMI, REBOOT, and BETAMI-DANBLOCK should not necessarily be interpreted as contradictory. Rather, they likely reflect differences in trial design, endpoint structure, and population characteristics within the broader context of modern post-MI care.

Certainly, the external validity of these findings appears reasonably strong, given that the major trials were conducted across diverse countries and healthcare systems. and in populations with heterogeneous STEMI/NSTEMI case mixes. The consistent lack of benefit across these settings, therefore, provides strong support for the absence of a clinically meaningful advantage once residual ischemia and adverse ventricular remodeling have already been attenuated by effective revascularization and guideline-directed medical therapy. A further consistent finding from contemporary randomized trials is that modern beta-blockers, when used at contemporary doses in post-MI patients, appear to be generally well tolerated. Rates of serious adverse events, including bradyarrhythmias, hypotension, or drug discontinuation, were relatively low across recent studies, supporting the overall safety of these agents in contemporary clinical practice. Both initiation of therapy, as observed in the REDUCE-AMI trial, and treatment withdrawal, as explored in the ABYSS trial, were not associated with deterioration in QoL [60, 61]. Evidence on beta-blocker withdrawal in patients with established HF also suggests that discontinuation should be approached with caution. Observational studies and secondary analyses of HF cohorts have shown that beta-blocker withdrawal or dose reduction is often associated with clinical deterioration or worse outcomes, particularly in patients with reduced LVEF or recent decompensation. Although these studies were not specifically designed to address post-MI populations, they highlight the potential risks of interrupting long-standing neurohormonal therapy and support a careful patient selection when considering treatment withdrawal [72, 73].

Several unanswered questions remain. The differential signals observed in BETAMI-DANBLOCK, particularly the reduction in re-MI, and the sex-specific findings reported in REBOOT should be interpreted as hypothesis-generating rather than practice-changing, given the lack of replication and limited statistical power for subgroup analyses. Likewise, the potential influence of beta-blocker class and dose, as well as interactions with age, sex, infarct type, and completeness of revascularization, remain incompletely defined and warrant further individual patient-data meta-analyses. More broadly, the clinical experience accumulated in HF populations indicates that withdrawal of guideline-directed neurohormonal therapies, including beta-blockers, should generally be considered only in carefully selected and clinically stable patients. Recent analyses across different HF cohorts further reinforce the importance of cautious deprescribing strategies, particularly in patients with residual ventricular dysfunction or ongoing arrhythmic risk [74, 75]. However, further evidence is needed in this respect to clarify the potential impact of beta-blockers in improved HF [76]. An additional aspect that deserves consideration concerns the applicability of these findings across specific high-risk clinical subgroups. Although the most recent randomized trials were conducted in multinational populations and reflect contemporary standards of care, the majority of enrolled patients were relatively stable and had undergone successful coronary revascularization with optimized secondary prevention therapy. As a result, the neutral effects observed in patients with preserved LVEF may not necessarily be generalizable to all post-MI populations. In particular, patients with incomplete revascularization, multivessel CAD, or residual ischemic burden may remain exposed to recurrent ischemic events and heightened sympathetic activation, conditions in which beta-blocker therapy may retain a protective role [77]. Similarly, individuals with significant arrhythmic substrate, including those with frequent ventricular ectopy or prior ventricular arrhythmias, may continue to derive benefit from the antiarrhythmic properties of beta-blockade [35]. The role of beta-blockers may also differ in elderly patients and in those with substantial comorbidities, in whom the balance between potential benefits (such as arrhythmia prevention and heart rate control) and adverse effects (including hypotension, bradycardia, or reduced exercise tolerance) may vary considerably [78]. Importantly, subgroup analyses from contemporary trials remain limited and often underpowered to detect clinically meaningful interactions across these populations. Therefore, until further dedicated analyses or trials are available, clinical decision-making should continue to integrate individual patient characteristics, ischemic burden, arrhythmic risk, and overall frailty when considering continuation or withdrawal of beta-blocker therapy. Beyond the role of beta-blockers, growing attention is being directed toward the potential impact of other guideline-directed medical therapies in the post-MI setting. Emerging evidence suggests that several components of contemporary HF pharmacotherapy may also influence ventricular remodeling and long-term outcomes after myocardial infarction, highlighting the need to interpret beta-blocker therapy within the broader framework of modern cardioprotective strategies [79, 80].

**Fig. 2:**
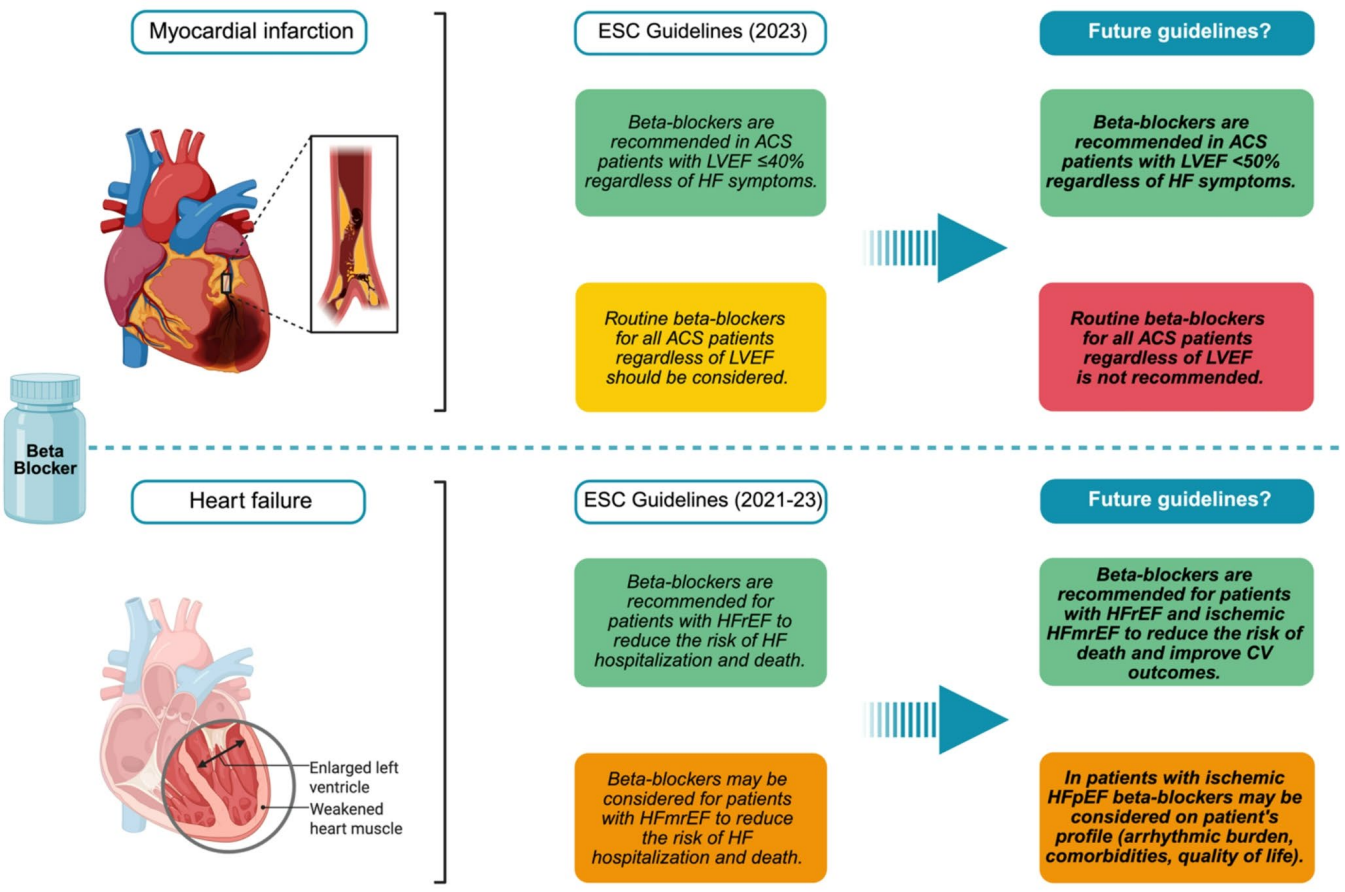
Current European guidelines on beta blocker therapy in acute coronary syndromes and heart failure followed by potential recommendations according to recent evidence. ACS: Acute coronary syndrome; CV: Cardiovascular; ESC: European Society of Cardiology; HF: Heart failure; HFmrEF: Heart failure with mildly reduced ejection fraction; HFpEF: Heart failure with preserved ejection fraction; HFrEF: Heart failure with reduced ejection fraction; LVEF: Left ventricular ejection fraction

In a clinical practice standpoint, recently updated ESC guidelines have maintained the recommendation of beta-blocker therapy in all patients with ACS, regardless of LVEF, and, in parallel, there is a weak recommendation for beta-blockers in HFmrEF, the but these guidelines were updated before the publication of recent and impactful evidence [10]. It is conceivable that future guideline updates may further refine current recommendations as additional evidence accumulates, potentially allowing a more nuanced and phenotype-oriented approach to beta-blocker therapy in patients with ischemic HF (Fig. 2). In this sense, the most important lesson of these trials may extend beyond beta-blockers themselves, reminding the CV community that even the most established therapies must periodically be re-evaluated in light of evolving evidence and contemporary clinical practice.

Taken together, these findings contribute to an evolving understanding of the role of beta-blocker therapy in ischemic HF and support the concept of a more tailored approach based on LVEF, clinical context, comorbidities, and patient tolerance [81]. Further evidence is needed to clarify whether specific clinical subgroups may derive differential benefit from continued therapy. Two ongoing randomized trials, the SMARTDECISION (NCT04769362) and the ABBREVIATE (NCT05081999), are specifically designed to compare discontinuation vs. continuation of beta-blocker therapy in ischemic HF with LVEF ≥ 40%, will provide further meaningful insights. The results of these trials will be essential to better define the optimal therapeutic strategy and to inform future guideline recommendations.

## Conclusions

**Fig. 3:**
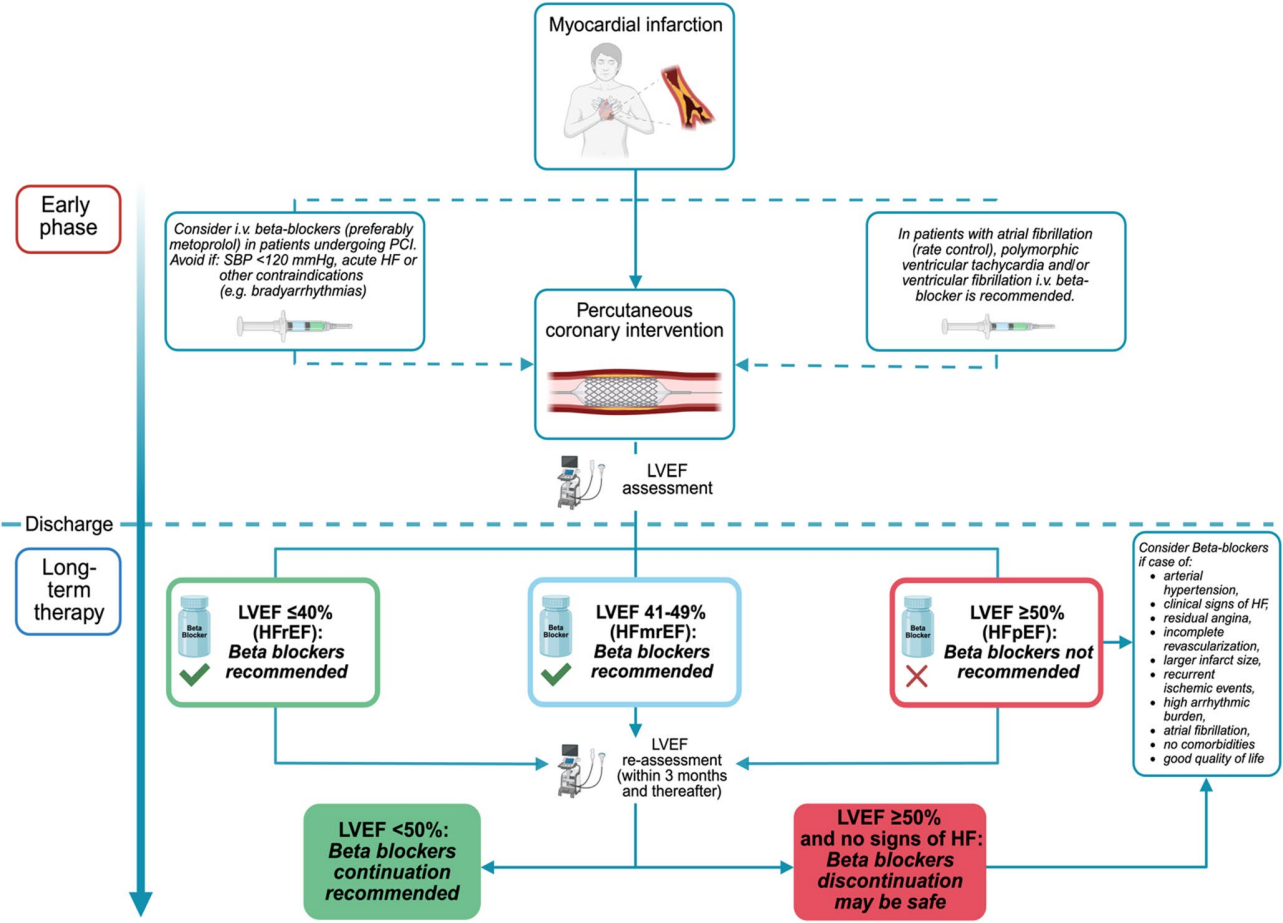
Conceptual algorithm summarizing the current evidence on the use of beta blockers in the acute phase of MI and in long-term treatment of ischemic HF. The proposed framework is hypothesis-generating and has not been prospectively validated. HF: Heart failure; HFmrEF: Heart failure with mildly reduced ejection fraction; HFpEF: Heart failure with preserved ejection fraction; HFrEF: Heart failure with reduced ejection fraction; IV: Intravenous; LVEF: left ventricular ejection fraction; PCI: percutaneous coronary intervention.; SBP: Systolic blood pressure

The beneficial impact of beta-blocker therapy in ischemic HF is well established in patients with reduced LVEF but is still controversial and unclear in those with mildly reduced and preserved LVEF. Taken together, the available evidence indicates that beta-blockers retain their greatest value in post-MI patients with reduced or mildly reduced LVEF, larger infarct size, incomplete revascularization, an arrhythmic substrate, or recurrent ischemic events. Conversely, in carefully selected low-risk patients, namely those with preserved LVEF (> 50%) and no prior or clinical sign of HF, available randomized evidence suggests that discontinuation of beta-blocker therapy may be safe, although caution is warranted in higher-risk subgroups such as patients with residual ischemia, significant arrhythmic burden, or complex coronary disease. For this reason, these findings should be interpreted cautiously until they are fully integrated into future guideline recommendations. By contrast, patients with HFmrEF should be pragmatically aligned with those with HFrEF and maintained on long-term beta-blocker therapy for secondary prevention. Importantly, reassessment of LVEF within 3 months after the index event may justify re-evaluation of beta-blockers use, considering the amount of stunned myocardium and the potential for recovery to preserved LVEF. Hence, we have proposed a conceptual therapeutic algorithm to illustrate how beta-blocker therapy might be individualized according to patient profile and clinical phase (Fig. 3). This framework derives from the synthesis of currently available evidence and should be regarded as hypothesis-generating, intended to stimulate future prospective validation on a more phenotype-oriented use of beta-blocker therapy in ischemic HF rather than to replace current guideline-directed management. Prospective validation could be achieved through pragmatic clinical trials or prospective registries evaluating algorithm-guided treatment strategies compared with standard guideline-based management.

Overall, these considerations support a more individualized and phenotype-oriented approach to beta-blocker therapy in ischemic HF, rather than a uniform treatment strategy applied to all post-MI patients. It is conceivable that advancements in contemporary medical and revascularization therapy might be responsible for the contemporary scarce association between beta-blocker treatment and improved survival in patients with MI. Nevertheless, further randomized trials are warranted to provide robust and evidence-based recommendations on the optimal strategy and timing of beta-blocker therapy in ischemic HF with the ultimate goal of improving outcomes of this population.

## Data Availability

No datasets were generated or analysed during the current study.
